# Differential impact of prostaglandin analogues on agreement of intraocular pressure measurements obtained by Goldmann applanation, rebound, and noncontact tonometry

**DOI:** 10.1186/s12886-021-02211-y

**Published:** 2021-12-17

**Authors:** Younhea Jung, Hyun Suh, Jung Il Moon

**Affiliations:** Department of Ophthalmology, Yeouido St. Mary’s Hospital, College of Medicine, The Catholic University of Korea, Seoul, Republic of Korea

**Keywords:** Prostaglandin analogue, Goldmann applanation tonometry, Noncontact tonometry, Rebound tonometry; intraocular pressure

## Abstract

**Background:**

To evaluate the effect of topical prostaglandin analogues on agreement of IOP measurements obtained by Goldmann applanation tonometry (GAT), rebound tonometry (RBT), and noncontact tonometry (NCT) in eyes with primary open- angle glaucoma (POAG).

**Methods:**

Intraocular pressure measurements were obtained using GAT, RBT, and NCT in patients with POAG with or without prostaglandin analogues. The agreement between each tonometry was analysed using Bland-Altman analyses in those with or without prostaglandin analogues. The effect of average IOP on IOP differences was also evaluated.

**Results:**

Among a total of 86 subjects included in the study, 44 patients were using prostaglandin analogues. The difference in IOP measured by GAT and RBT was marginally greater in those with (GAT-RBT: − 0.94 ± 1.63 mmHg) prostaglandin analogues than in those without (− 0.33 ± 1.22 mmHg, *P* = 0.06). The difference in IOP measured by GAT and NCT was significantly greater in the prostaglandin group (GAT-NCT: 2.40 ± 2.89 mmHg) than in the group without prostaglandin analogues (0.41 ± 1.63 mmHg, *P* < 0.01). While there was no significant relationship between the average of all tonometries and the difference between tonometries in those without prostaglandin analogues, both RBT and NCT underestimated IOP relative to GAT at higher IOP in those using prostaglandin analogues.

**Conclusion:**

Intraocular pressure measured by RBT and NCT was similar to that measured by GAT in those without prostaglandin analogues. RBT overestimated and NCT underestimated IOP compared to GAT in those using prostaglandin analogues.

## Background

Intraocular pressure (IOP) is the most important risk factor in the pathogenesis of glaucoma; therefore, the accuracy of its measurement is mandatory for the diagnosis and management of glaucoma [1–4]. While Goldmann applanation tonometry (GAT) is considered the gold standard technique for measuring IOP, rebound tonometry (RBT) and noncontact tonometry (NCT) are also widely used in clinical settings because they are convenient and do not need local anaesthesia. A number of studies have evaluated the agreement among these tonometries. When comparing RBT and GAT, most studies detected IOP measurements by RBT higher than GAT [5–8]. Several studies have compared NCT and GAT and have reported more heterogeneity in the results. Some studies found IOP measurements by NCT to be higher than those by GAT [5, 9], while other studies have found NCT measurements to be lower than those by GAT [6].

Measurements of IOP by these devices are obtained indirectly and are based on several assumptions about the cornea, including its biomechanical properties [10]; thus, factors that alter the biomechanical properties of the eye can affect the accuracy of IOP measurements. In addition, GAT, RBT, and NCT are based on different principles, and hence may have differential impacts from changes in biomechanical properties of the cornea.

Topical prostaglandin analogues have been previously reported to alter the biomechanical properties of the eye [11–14]. Prostaglandin analogues are often chosen as first-line drugs in the treatment of glaucoma because of their high efficacy and few systemic side effects [15, 16]. They decrease IOP by increasing uveoscleral outflow. Previous studies have shown that prostaglandin analogues degrade extracellular matrix in the ciliary muscle by upregulating matrix metalloproteinases and downregulating tissue inhibitors of matrix metalloproteinases [17, 18]. They also affect the extracellular matrix of the cornea, which could alter its biomechanical properties [11–14].

To the best of our knowledge, the effect of topical prostaglandin analogues on IOP measurements using GAT, RBT, and NCT has not been previously reported. Therefore, the objective of our study was to determine the effect of topical prostaglandin analogues on agreement of IOP measurements obtained by GAT, RBT, and NCT in eyes with primary open-angle glaucoma (POAG).

## Methods

The study was approved by the Institutional Review Board (IRB) of Yeouido St. Mary’s Hospital and adhered to the tenets of the Declaration of Helsinki. In this retrospective study, informed consent was waived by the IRB, because the data were analysed anonymously.

Patients were recruited at the glaucoma clinic at Yeouido St. Mary’s Hospital, College of Medicine, The Catholic University of Korea between August and October 2019. Patients older than 40 years with an established diagnosis of POAG using at least one antiglaucomatous eyedrop were consecutively included in the study. The diagnosis of POAG was made by a glaucoma specialist based on the following criteria: open angle on gonioscopy, normal anterior chamber, glaucomatous optic disc (localized or diffuse neuroretinal rim loss, excavation, or retinal nerve fibre layer defect) on dilated fundoscopy, and an abnormal visual field consistent with glaucoma (less than 20% fixation loss, less than 15% false-positive error, and less than 15% false-negative error) on at least two consecutive tests. Patients with corneal diseases, corneal astigmatism >3D, previous refractive surgery or keratoplasty, intraocular surgery within the previous 3 months, or tight orbit syndrome were excluded from the study.

Patients underwent a complete ophthalmic examination including IOP measurements using GAT (Haag-Streit, Switzerland), RBT (ic200, Icare, Finland), and NCT (CT-80, Topcon, Japan), central corneal thickness by ultrasound pachymetry (Ultrasonic Scanner, Tomey, Japan), and axial length measurement (IOLMaster 500, Carl Zeiss Meditec AG, Germany). IOP measurements were obtained sequentially within 30 min with at least 5 min between each measurement in the following order to avoid the influence of previous measurements: NCT, RBT, and GAT. For each tonometry, an average of 3 measurements was used. Three examiners independently performed each tonometry, and each examiner was blinded to previous IOP measurements. For RBT measurement, each IOP measurement consisted of 6 measurements that were averaged automatically. If both eyes were eligible for inclusion, only one eye from each patient was randomly selected for analyses.

Independent t-tests and chi-square tests were used to compare continuous and categorical variables, respectively. The mean IOP from RBT, NCT, and GAT were compared using one-way repeated-measures analysis of variance, followed by Bonferroni correction to adjust the *P* values for multiple comparisons. In addition, Bland-Altman plots were used to display the mean ± 2 SD disagreement between the 2 selected IOP measurements. In the Bland-Altman graphs, the difference between each IOP measurement was plotted against the mean of the 2 measurements. A linear regression model was used to calculate the relationship between the average IOP of all tonometries and the IOP difference between measurements. Statistical analyses were performed using SPSS (ver. 17.0; SPSS Inc., Chicago, IL). A *P* value < 0.05 was used to indicate statistical significance in all analyses.

## Results

Among a total of 86 subjects included in the study, 38 were male and 48 were female; 44 patients were using prostaglandin analogues, and 42 patients were not. The mean duration of prostaglandin analogue usage was 57.76 months (Table 1).

**Table 1:** Clinical characteristics of study subjects

*N* = 86	Without prostaglandin analogue (*N* = 44)	With prostaglandin analogue (*N* = 42)	*P* value
Age (years)	66.67 ± 5.79	66.98 ± 10.83	0.88
Gender, male/female	20/24	18/24	0.81
Spherical equivalent	−2.18 ± 3.41	−2.31 ± 3.37	0.86
Central corneal thickness (μm)	547.23 ± 21.12	544.44 ± 50.85	0.79
Axial length (mm)	23.31 ± 2	23.44 ± 1.61	0.85
Duration of prostaglandin analogue (months)	–	57.76 ± 30.81	

**Fig. 1:**
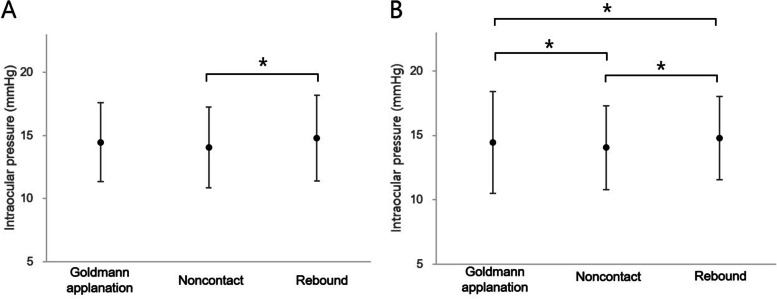
Mean intraocular pressure measured by Goldmann applanation tonometry, rebound tonometry, and noncontact tonometry in those without prostaglandin analogues (**A**) and those with prostaglandin analogues (**B**)

All IOP measurements are shown in Fig. 1. In those without prostaglandin analogues, no significant differences were found between mean IOP measured by GAT (14.45 ± 3.12 mmHg) and RBT (14.79 ± 3.39 mmHg, Bonferroni post hoc test *P* = 0.24) and by GAT and NCT (14.05 ± 3.17 mmHg, Bonferroni post hoc test *P* = 0.31). There was a statistically significant difference in mean IOP between RBT and NCT measurements (Bonferroni post hoc test *P* = 0.03). In the prostaglandin group, there were significant differences in the mean IOP between GAT (17.35 ± 3.96 mmHg) and RBT (18.29 ± 3.25 mmHg, Bonferroni post hoc test *P* < 0.01), GAT and NCT (14.95 ± 3.27 mmHg, Bonferroni post hoc test *P* < 0.01), and RBT and NCT (Bonferroni post hoc test *P* < 0.01).

**Fig. 2:**
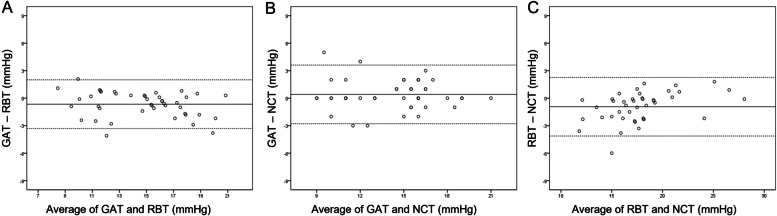
Bland–Altman plots comparing the agreement of intraocular pressure between GAT and RBT (**A**), between GAT and NCT (**B**), and between RBT and NCT in patients without prostaglandin analogues. Middle line: mean difference; upper and lower lines: 95% limits of agreement. GAT: Goldmann applanation tonometry, RBT: rebound tonometry, NCT: noncontact tonometry

**Fig. 3:**
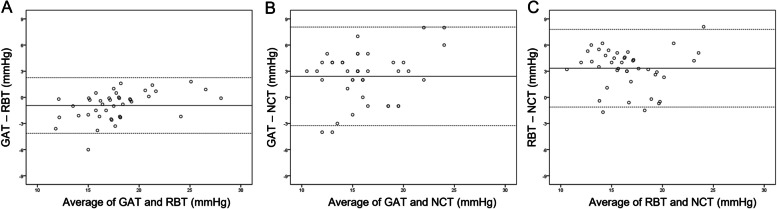
Bland–Altman plots comparing the agreement of intraocular pressure between GAT and RBT (**A**), between GAT and NCT (**B**), and between RBT and NCT in patients with prostaglandin analogues. Middle line: mean difference; upper and lower lines: 95% limits of agreement. GAT: Goldmann applanation tonometry, RBT: rebound tonometry, NCT: noncontact tonometry

Figure 2 shows Bland–Altman scatterplots comparing IOP between each tonometry in patients without prostaglandin analogues. The values of the 95% levels of agreement were − 3.31 to + 2.00 mmHg (GAT-RBT), − 2.79 to + 3.61 mmHg (GAT-NCT), and − 2.88 to 5.02 mmHg (RBT-NCT). In those with prostaglandin analogues, the values of the 95% levels of agreement were − 4.13 to + 2.26 mmHg (GAT-RBT), − 3.25 to + 8.06 mmHg (GAT-NCT), and − 1.11 to + 7.79 mmHg (RBT-NCT) (Fig. 3).

Table 2 shows a comparison of the differences in IOP measured by GAT, RBT, and NCT between those with and without prostaglandin analogues. The difference in IOP measured by GAT and RBT was marginally greater in those with (− 0.94 ± 1.63 mmHg) prostaglandin analogues than in those without (− 0.33 ± 1.22 mmHg, *P* = 0.06). The difference in IOP measured by GAT and NCT was significantly greater in the prostaglandin group (2.40 ± 2.89 mmHg) than in the group without prostaglandin analogues (0.41 ± 1.63 mmHg, *P* < 0.01).

**Table 2:** Comparison of difference in intraocular pressure measured by GAT, RBT, and NCT

*N* = 86	Without prostaglandin analogue (*N* = 44)	With prostaglandin analogue (*N* = 42)	*P* value
GAT – RBT (mmHg)	−0.33 ± 1.22	−0.94 ± 1.63	0.06
GAT – NCT (mmHg)	0.41 ± 1.63	2.40 ± 2.89	< 0.01
RBT - NCT (mmHg)	0.74 ± 1.85	3.34 ± 2.27	< 0.01

*GAT* Goldmann applanation tonometry, *RBT* Rebound tonometry, *NCT* Noncontact tonometry

**Fig. 4:**
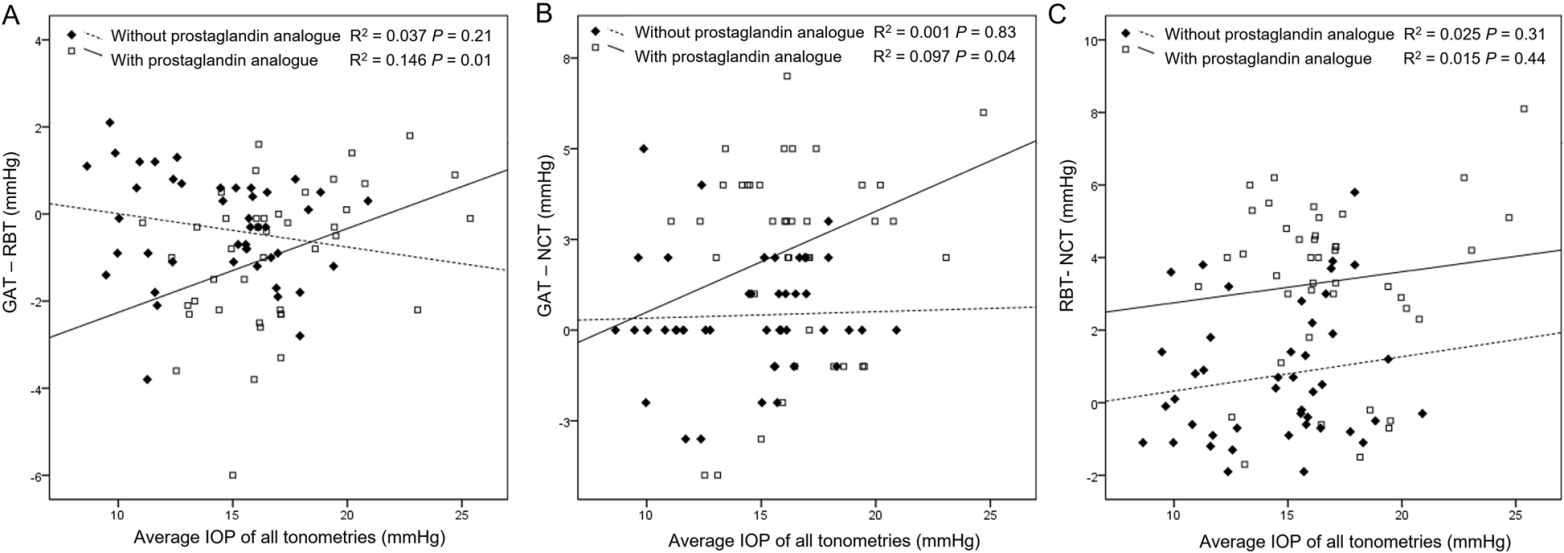
The effect of average IOP of all tonometries on IOP differences between GAT and RBT (**A**), between GAT and NCT (**B**), and between RBT and NCT. GAT: Goldmann applanation tonometry, RBT: rebound tonometry, NCT: noncontact tonometry

There was no significant relationship between the average of all tonometries and the difference between tonometries in those without prostaglandin analogues (Fig. 4). However, both RBT and NCT underestimated IOP relative to GAT at higher IOP in those using prostaglandin analogues.

## Discussion

In this study, we found that RBT overestimated and NCT underestimated IOP compared to GAT in those using prostaglandin analogues, whereas IOP measured by RBT and NCT were similar to that measured by GAT in those without prostaglandin analogues. In addition, the difference between tonometries was significantly greater in those with prostaglandin analogues than in those without.

The Bland–Altman scatterplots showed overall good agreement between tonometries in those without prostaglandin analogues. The mean differences and limits of agreement were − 0.66 ± 2.66 mmHg (GAT-RBT), 0.41 ± 3.20 mmHg (GAT-NCT), and 1.07 ± 3.95 mmHg (RBT-NCT). In patients using prostaglandin analogues, the Bland–Altman scatter plots showed less agreement between tonometries: the mean differences and limits of agreement were − 0.94 ± 3.20 mmHg (GAT-RBT), 2.40 ± 5.66 mmHg (GAT-NCT), and 3.34 ± 4.45 mmHg (RBT-NCT), respectively. Furthermore, while there was no significant relationship between the average of all tonometries and the difference between tonometries in those without prostaglandin analogues, both RBT and NCT underestimated IOP relative to GAT at higher IOP in those using prostaglandin analogues.

These findings suggest that while all tonometers would be clinically acceptable, careful attention should be given to those using prostaglandin analogues. The use of topical prostaglandin analogues has been shown to alter the biomechanical properties of the cornea, thereby affecting the accuracy of IOP measurements [11, 14, 19]. Topical use of prostaglandin analogues alters the balance between matrix metalloproteinases and tissue inhibitors of matrix metalloproteinases, resulting in degradation of corneal extracellular matrix [12]. They have also been shown to alter the biomechanical properties of the cornea in vivo. Meda et al. [11] reported that chronic use of prostaglandin analogues induced changes in biomechanical properties of the cornea, which contributed to inaccurate measurement of IOP. Bolivar et al. [19] also reported that treatment with topical prostaglandin analogues increased corneal hysteresis irrespective of the IOP decrease, suggesting its direct effect on corneal biomechanical properties.

The biomechanical properties of the cornea have been previously reported to have a greater influence on IOP measurement than central corneal thickness [20]. The effect of corneal biomechanical properties on IOP differences measured by various tonometers has been shown in a few previous studies [21–23]. The difference in IOP measured by GAT and NCT was greater in those with low corneal hysteresis in normal controls [23]. Another study also found that corneal viscoelasticity measured by corneal hysteresis and corneal resistance factor affected IOP differences between GAT and NCT [22]. Shin et al. [21] reported that RBT underestimated corneal-compensated IOP measured by Ocular Response Analyzer (ORA) in glaucoma patients with lower corneal hysteresis, but not in normal controls. They speculated that the impact of corneal biomechanical properties on IOP measurements may be greater in glaucoma eyes than in normal eyes. Prostaglandin analogues have been reported to increase corneal hysteresis, [19, 24] so our present findings are in general agreement with their study.

Although the mechanism underlying greater differences in IOP measured by various tonometers in those with prostaglandin analogues is unclear, the differential impact among tonometers may be related with different corneal surface areas flattened by each tonometer. Goldmann applanation tonometry measures the force necessary to flatten the corneal surface using a tip with a diameter of 3.06 mm. A noncontact tonometer uses an air impulse to flatten the cornea surface. The IOP is calculated by an optoelectronic applanation monitoring system that senses light reflected from the corneal surface [25]. Rebound tonometry uses a magnetized probe, which is 0.3 mm in diameter with a plastic end tip 1.7 mm in diameter, and calculates the IOP based on its deceleration after bouncing off the cornea [26].

Similar to our results, a previous study by Sanchez-Barahona et al. [27] compared the IOP reductions measured by GAT, ORA, and Corvis ST tonometry in patients with latanoprost and showed that the ocular hypotensive effect of prostaglandin analogues was different when measured using GAT or ORA compared to Corvis ST. However, they did not compare those with prostaglandin analogues and those without.

Our study has several limitations. First, we did not have evidence of changes in corneal biomechanical properties in this study. The duration of prostaglandin analogue administration could also have affected the changes in corneal biomechanical properties. Future studies are warranted in this regard. Second, we lack longitudinal data regarding IOP measurements, which may yield interesting results. Third, our data showed a significant difference in IOP between RBT and NCT even in patients without prostaglandin analogues, so one could argue that our findings are affected by the limited reproducibility of tonometers. We measured each IOP multiple times, and it has been reported that the difference in IOP measured by various tonometers may be greater in eyes with glaucoma; therefore, we assume that this had a minor effect on our findings [21]. In addition, all patients in the study population were Asian; therefore, it is difficult to generalize our findings to other ethnicities.

## Conclusions

In conclusion, we showed that patients with prostaglandin analogues showed greater disagreement between various tonometries. These findings warrant caution when clinicians use different tonometries to measure IOP in these patients.

## Data Availability

Due to ethical restrictions, data are available upon request from the corresponding author for researchers who get approval from Yeouido St. Mary’s Institutional Data Access Committee (http://cmccrcc.catholic.ac.kr/english/main.jsp).

## Abbreviations

GAT: Goldmann applanation tonometry
IOP: Intraocular pressure
NCT: Noncontact tonometry
ORA: Ocular response analyzer
RBT: Rebound tonometry
